# Collaborative Working Architecture for IoT-Based Applications†

**DOI:** 10.3390/s18061676

**Published:** 2018-05-23

**Authors:** Higinio Mora, María Teresa Signes-Pont, David Gil, Magnus Johnsson

**Affiliations:** 1 Department of Computer Science Technology and Computation, University of Alicante, 03690 Alicante, Spain; teresa@dtic.ua.es (M.T.S.-P.); dgil@dtic.ua.es (D.G.); 2 Department of Intelligent Cybernetic Systems, NRNU MEPhI, 115409 Moscow, Russia; magnus@magnusjohnsson.se; 3 Department of Philosophy, Lund University Cognitive Science, 22362 Lund, Sweden; 4 Magnus Johnsson AI Research AB, 24334 Höör, Sweden

**Keywords:** embedded systems, internet of things, mobile cloud computing, computer modelling, sensor processing modeling

## Abstract

The new sensing applications need enhanced computing capabilities to handle the requirements of complex and huge data processing. The Internet of Things (IoT) concept brings processing and communication features to devices. In addition, the Cloud Computing paradigm provides resources and infrastructures for performing the computations and outsourcing the work from the IoT devices. This scenario opens new opportunities for designing advanced IoT-based applications, however, there is still much research to be done to properly gear all the systems for working together. This work proposes a collaborative model and an architecture to take advantage of the available computing resources. The resulting architecture involves a novel network design with different levels which combines sensing and processing capabilities based on the Mobile Cloud Computing (MCC) paradigm. An experiment is included to demonstrate that this approach can be used in diverse real applications. The results show the flexibility of the architecture to perform complex computational tasks of advanced applications.

## 1. Introduction

The new era of Knowledge Society has brought advanced services for improving the quality of life of citizens and making better use of resources. These services are based on modern paradigms of Information and Communication Technologies (ICT) such as the Internet of Things (IoT) and the Cloud Computing paradigms. In this way, new concepts have been created which apply IoT to benefit different areas of society and industry. For instance: ambient assisted living [1], smart cities [2] or smart logistics [3]. In these scenarios, new data management issues arise for integrating environmental sensor data efficiently and handling data from different sources [4].

Recent applications have been developed around the aforementioned concepts where sensing and processing capabilities of the devices play an important role. These devices are usually embedded systems and/or mobile devices such as smart phones, wearables, laptops, tablet PCs, etc. To deploy complex artificial intelligence applications in IoT environments provides a powerful driver for increased edge computing capabilities. Real-world use cases of artificial intelligence combining with the Internet of Things [5]. This evolution promotes a digital transformation of the society by providing the citizens and professionals with advanced applications for sensing and analyzing data on the ground. Due to the recent successes and the anticipated breakthroughs in different fields, it has now become one of the most promising research areas [6]. Indeed, this fact is largely accelerated by new smartphones and communication capabilities. However, the design of advanced IoT-based applications remains a challenge [7]. Handling simultaneous data flows, data processing and/or complex mathematical function execution could overflow the computing capabilities of the embedded systems and mobile devices.

One approach to overcome this drawback consists in designing a distributed system where the sensor devices are the distributed part to acquire the data and a centralized infrastructure that performs the hard processing. The classical client/server architecture has been designed for that purpose. Currently, this centralized infrastructure is usually deployed in the Cloud [8]. However, this shift introduces several new risks, and some bottlenecks and delays may result from the communications among the devices and the centralized system. In particular, the latter drawback is strongest for multimedia data, for example, in applications that use video and image acquisition devices. For that reason, it is difficult to implement a centralized multimedia analysis system in the cloud [9,10].

To overcome these bottlenecks and delays, this work extends our recent proposal of a distributed architecture [11] to perform collaborative work for IoT-based environments and sharing the application workload among the available devices. This improved architecture takes into account the different network layers and their computing platforms involved, from the remote Cloud servers to connected smart sensors and “things”. The approach aims at optimizing the use of computational resources of an IoT environment while providing a framework able to obtain data from sensors, perform complex computational tasks and run advanced applications.

Our *working hypothesis* is that the conception and development of processing models based on schemes of collaborative working and cloud computing can supply the necessary processing power for running applications when they run on embedded devices with limited performance. The auxiliary use of cloud computing infrastructure on demand will provide flexibility to perform the necessary tasks as well as mechanisms to support the service quality maintenance. This must be accomplished even with devices and sensors with low computing capability. The *main contribution* of the paper is the proposal of a distributed architecture that combines sensing and processing at different levels of the network to perform a collaborative work based on the Mobile Cloud Computing (MCC) paradigm.

This approach can be used in a diversity of real applications running in different environments under different conditions where a set of computing systems are available. To validate the proposal, this research includes a set of experiments with an open dataset split into several subsets to enable parallel testing. The main goal of this splitting is to prove that the collaborative work does affect neither the final accuracy nor the rules and knowledge obtained.

The rest of the paper is organized as follows: Section 2 describes the basic background of distributed computing for IoT; Section 3 defines the collaborative working architecture; Section 4 describes the validating experiments; and finally, Section 5 draws the relevant conclusions and presents future work.

## 2. Background of IoT Distributed Computing

Nowadays it is very common that, due to the high performance of the servers, there is not a strong need of taking the time to design a good plan where all the resources are used. However, it is a reality that smart devices come continuously with greater computing capabilities, and moreover, other conditions can exist that prevents the use of external cloud servers.

Distributed computing is a field of computer science studying distributed systems. A distributed system can be of a variety of possible configurations, and its components can be of different size and performance such as servers, workstations, personal computers, mobile computers, smart devices and smart sensors. The goal of distributed computing is to make such a network work in a collaborative way, that is, run as a single system.

In this way, the components of a distributed system are located on networked computers, communicating and coordinating their actions by means of exchanging messages to meet a common goal [12,13].

In addition to the existing variety of embedded systems, the mobile devices, such as smartphones, tablets, laptops, wearables, etc., play an important role in the development of IoT solutions. Mobile devices have evolved considerably in recent years as a result of improvements in mobile technology, mobile networking and mobile computing. Examples of such improvements are increased processing power of mobile devices, novel forms of user interaction and new connectivity protocols. Hardware improvements have motivated the explosive growth of mobile applications, especially those that can perform off-screen processing, reducing battery consumption. These processing features are commonly adopted by mobile applications or ‘Apps’ such as music players and activity trackers that are widespread in the smartphone user community. Nowadays, the rapid expansion of smartphone ownership across the world reaches 80–90% of penetration rate in the most advanced markets [14]. This trend places in the hands of users a powerful tool for accessing to the Cloud services and IoT resources to run sophisticated applications. In this way, significant IoT opportunities are starting to emerge for citizens.

The IoT environments are highly distributed architectures and they can be considered as very weakly-coupled computer systems. Usually, the connected devices are heterogeneous and present a dynamic behaviour.

In the view of the recent works on this topic, there are four major research lines in distributing application systems for IoT environments that are transversal areas in the design of many advanced applications such as Artificial Intelligence applications, games, e-business and e-commerce, etc. These lines are the following: (i) framework design for distributed computing, (ii) integration with Cloud Computing resources or MCC concept, (iii) security, and (iv) distributed applications design. There are a lot of research focused on all these topics. The next subsections describe the recent works on these issues. Table 1 summarizes some relevant and representative contributions.

### 2.1. Framework Design for Distributed Computing

The framework is basically related to modelling the distributed system, the communication protocols, access and discovery services and the design of the scheduling method of the tasks along the whole system. This issue may include the specification of the devices involved and the requirements of the system operation such as the Quality of Service (QoS) [15,16], the definition of task and the application constraints [17,18]. As a representative example, a distributed framework based on IoT is proposed in [19] for monitoring human biomedical signals in activities involving physical exertion. The major advantages and novelties of this scheme is the flexibility in computing the health application by using resources from available devices inside the body area network of the user.

Other approaches develop the concept of Virtual Sensor as an abstraction of real sensors, reproducing their logical behaviour and enriching their functionalities with programmable operators [20,21]. The use of virtual sensors has increased continuously for translating the passive sensors to smart things and to simplify creating and configuring complex applications. This evolution enables better sensor management capability and facilitates the distribution of data and computations among them.

New middlewares for distributed environments aim to improve the integrated management of heterogeneous data, resources and events as well as to provide resource discovery and interoperability within the diverse applications and services. Recently, there have been a number of proposals for middlewares tackling with several aspects of IoT distributed systems [22].

Finally, recent trends are pushing the processing capabilities to the edge of network where data analytics and knowledge generation are performed by heterogeneous things [23]. This approach achieves a dramatic reduction in latency and ensures the security of locality information [24]. Regarding to this issue, intensive research is carried out to adapt the job-based scheduling methods to embedded devices and types of applications involved in the IoT environments to fully utilize the nodes and to achieve performance improvements [15,25,26,27,28].

### 2.2. IoT and Cloud Computing Combination

The cloud computing is considered as resource provisioning paradigm in distributed systems. In this way, the cloud infrastructure can centralize most part of the processing cost. The combination of IoT and Cloud Computing generates synergy for both paradigms and makes the objects smarter [29]. Cloud-based intelligence provides value to IoT systems since datacenters can compute the complex machine learning and big data processing methods to infer meaning from the raw data [5]. This integration called CloudIoT allows to provide powerful applications to the users in several real-world domains [30,31,32,33].

However, a system can only handle so much data before the system slows down and latency grows to an unacceptable point for the application. Recent approaches try to leverage the computing power of the devices and to outsource the workload to the cloud only when necessary. This trend is called Mobile Cloud Computing (MCC). This paradigm is a promising way to improve the performance as well as reducing the power consumption of a “thing” by executing some parts of the application on a remote server. This combines cloud computing, mobile computing and wireless networks to bring rich computational resources to mobile users, network operators, as well as cloud computing providers [34,35]. In this way, the distribution of the processing between devices of the IoT and cloud computing resources is able to increase the capabilities of the IoT system and to achieve greater overall performance in application execution. There are several techniques to improve the efficiency and effectiveness of the offloading process, such as multi-criteria decision analysis [36], stochastic analysis [37], or application-oriented [38].

The efficient dynamic allocation of tasks is a very important and difficult topic on IoT environments. Sometimes synchronization between the server-side layer and the devices or between devices is needed. In this regard, push notifications are the delivery of information from a software application to a computing device without a specific request from the device. They originate from the server-side layer and they are used to receive processing alerts in the device. An important advantage of push notifications in MCC is that the technology does not require that device’s Apps are open in order to receive these alerts [39]. This allows smartphones and wearables to receive and display text message alerts even when their screens are locked and the app that receives the push notification is closed.

### 2.3. Security

Presently, the security is one of the most restrictive concerns for technological development in areas such as IoT or Cyber-Physical Systems [40]. This issue gains even more importance when the processing load is shared with cloud resources [41] and MCC paradigm [42]. The most important aspects in these fields are guaranteeing user privacy, data confidentiality and the provision of application security that uses cloud resources. These requirements play an essential role as nowadays IoT is characterized by heterogeneous technologies, which concur to the provisioning of innovative services in various application domains [43]. Due to this complexity of the nature of distributed IoT paradigm it is very challenging to design fully secure methods and protocols to detects and prevents security vulnerabilities and attacks [44].

In addition, the new European General Data Protection Regulation (GDPR) [45] imposes restrictive requirements to data processing, especially when the data come from human monitoring. GDPR compliance is particularly challenging in IoT environments, because it can be difficult to gain the consent needed to process the personal data. Therefore, a GDPR awareness stage is needed in the IoT system design process to analyze its compliance taking into account the technologies involved and the processing made [46]. User privacy challenges arise even when generating information or inferences about the user behaviour [47].

However, security and privacy can also be improved with IoT distributed systems. The GDPR prohibits the transfer of data to countries that don’t have an adequate level of data protection [48]. Currently, cloud providers can deploy their datacenters infrastructure out of ‘safe’ regions. In this way, the collaborative work among the objects helps protect the sensitive data by keeping it at the source rather than sending that information to the cloud [49].

In addition, the distributed nodes can be used for enhancing the overall security of the network. Therefore, there are works that propose to distribute the security checking workload over the network and/or a cloud computing server. There exist multiple options in relation to what network nodes should be responsible for the execution of the security processes [50].

### 2.4. Distributed Applications Design

The applications for IoT are essentially distributed systems by nature. In this way, IoT is becoming an emerging Internet-based information architecture to allow data and information flows from the real world to advanced users and industrial applications.

Application design relies heavily on thing programming close to the operating system level, which reduces efficiency and reliability of the IoT application [51]. The design complexity is reduced significantly, when using web services [52].

There are several relevant works to update knowledge and current challenges. For example, the research presented by [53] introduces a cloud-based platform for the deployment of distributed IoT applications. The main characteristics of this platform are that each object is an autonomous social agent; the platform as a service (PaaS) model is fully exploited; reusability at different layers is considered; the data is under control of the users. There are also commercial frameworks and platforms designed for developing and running Internet of Things applications [54]. The importance of this platforms makes it possible to develop standards-based endpoints and data warehousing, that will enable secure interoperation of modularized and distributed applications. These solutions can also support big data processing and runtime autonomic management [55].

The feature of reusability is very significant as it allows the programmers to generate templates of things and their services. Connected things may use different types of protocols and connectivity patterns. In this way, design patterns add an abstract layer and facilitate to make designs robust and reusable solutions [56].

Distributed applications classically record a numerous set of events, messages and log files, making these platforms an excellent data source for process mining tools [57]. Nevertheless, understanding the behaviour of all these applications, very often with heterogeneous devices, from their event logs can be a complex as well as error prone task. In this area, the rapid advances in interoperability and information integration methods have driven massive growth in the use of integrated information systems for IoT applications [58].

Finally, centralized approaches have been the prevailing choice for providing intelligence based on the acquired data. But, beyond response times and network loads, some distributed applications need a decentralized intelligence approach in order to better meet the environment and application requirements. There are proposals to move intelligence to the edge in order to offer low-level intelligence for IoT applications [59]. In this line, decentralized multiagent systems provides ways of dealing with autonomy and heterogeneity [60]. In addition, this approach enables discovery and selection of IoT devices and data resources.

### 2.5. Findings

After reviewing the representative proposals in this field, some findings can be identified that justify and summarize our contributions to previous works:

- The number of connected things is increasing significantly. This increases the possibilities of designing advanced applications that take advantage of their ubiquitous sensing and computing possibilities.
- The computing resources of the whole network can be used for enhancing the performance of IoT-based applications by sharing the processing load among the available platforms, and a way to leverage more intensively the deployed infrastructure.
- Despite the progress achieved by recent research, the proper distribution of the application workload remains a challenge. There is a lack of formalization and commonly agreed mechanisms to implement real collaborative applications for IoT environments.

This work fits perfectly with Framework design for distributed computing. In this area, the aforementioned findings lead us to advance continuously in the development of new frameworks and architectures. Basically, the contributions presented in Table 2 summarize the research line in different topics. This is a challenging issue, and therefore, progresses are slowly made. Most of the proposals are focused on a specific topic such as modelling [18,19], quality of experience [16], edge of things [24], middleware [22,23], and scheduling method [17,25,26,27,28].

In this line, the research presented represents a step forward in designing collaborative schemes of IoT applications by sharing the application’s workload between the IoT devices of the environment. A new application formalization and scheduler module is introduced to handle the working collaboration among heterogeneous things and other networked resources. The components of the scheduler are detailed and a practical use case is described.

## 3. Distributed Computational Architecture

### 3.1. General Scheme

The primary objective of the proposed distributed architecture is to take advantage of the deployed infrastructure of things and the cloud computing resources to reduce the computing costs and improve the overall performance. The main idea is to share the application’s workload between the server-side and the rest of things with computing capabilities such as smartphones, wearables, tablets, smart sensors, and other embedded devices. This workload-sharing among the things enables a horizontal scaling to mitigate costs, rather than resort to remote servers. Thus, in accordance with our proposal, these kinds of devices perform more processing tasks than the server-side layer. In addition, cloud computing is available to use only as a last resort if needed. In the case of the asynchronous synchronization needs between cloud server computing and the different devices, our system develops a push notification-based approach.

In this section, a model of computation suitable for IoT applications is defined according to that architecture. The proposal focuses on distributed applications that can be represented by a graph 𝔸 = {𝕌, 𝔽} where:

- 𝕌 <vertex> represents the execution units of the application. Therefore, the IoT application can be broken down into a list of execution units: 𝕌 = {u_0_, u_2_, …, u_n−1_}.
- 𝔽 <edge> represents the data flows exchanged between the execution units. The data flows set the precedence between the execution units and the volume of exchanged data. F(i,j) ∈ 𝔽 defines the volume of data exchanged between the execution unit i and j.

**Figure 1:**
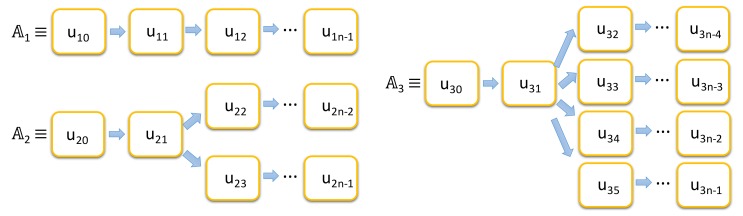
Diagram of different flow possibilities in our proposed distributed system.

The execution units of an application are related to its capacity of processing data and tasks in parallel. It is a very important feature for modern machine learning and big data approaches on IoT applications, since the edge things can increase significantly the performance and costs of the system without having to send the data to the server for a centralized processing. For example, Figure 1 shows three cases of applications (𝔸_i_) modeled according to this principle where the fragmentation feature of data generates more execution units and opens more processing opportunities among the things.

In accordance with this model, the devices ⅅ involved in the IoT applications are defined as follows:

- Let S be the set of sensor devices. These devices do not have computing capabilities themselves. Their work consists in sensing and communicating the data to other devices or the cloud.
- Let P be the set of available computing platforms. This set includes the things that have processing capabilities. The devices of the P set can also acquire the data and process it.
- Let C be the set of cloud computing resources. In this set the remote servers where the processing load is outsourced are located.
  That is: ⅅ = {S} ⋃ {P} ⋃ {C}

**Figure 2:**
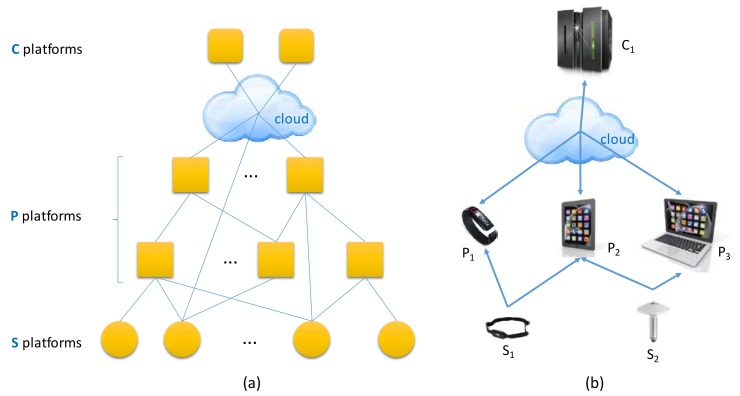
IoT communication network. (**a**) General scheme; (**b**) Example case.

The elements of those sets are interconnected creating the IoT communication network. The architecture model distributes execution units {𝕌} across the available devices ⅅ according to their particular capabilities and the application constraints. Figure 2 illustrates this idea.

The IoT environments have a dynamic behaviour since new things can appear and disappear. Therefore, a discovery service of things is needed to conduct the registration of a new device and unregistration when it is not available. This service builds the set ⅅ and, therefore, it plays a significant role in design of IoT applications as they can allow clients and applications to access available resources and data provided by things. This discovery service can be centralized [61] and/or decentralized [62]. There are several proposals in the literature focused on this service [63,64]. In this work, it is supposed that a proper discovery service is running to maintain the set ⅅ updated.

To distribute resources in an efficient way and meet the application requirements, the resource utilization must be properly characterized. For this reason, a vector 𝕍 is proposed defined as a vector domain of relevant features modelling the behaviour of the computing load on each device. For applications such as the ones this work focuses on, 𝕍 could be defined as a domain of vectors with two components in the range [0, 1], with the following semantics:

𝕍 = Response_time × Transfer_rate.(1)

The “Response_time” of a computer is defined proportionally to the execution time required by a selected benchmark execution unit in the computer (0 and 1 are mapped to situations where the processor is practically idle and unacceptably busy, respectively). The benchmark task does not communicate through the network, so it does not take into account network latencies, but only local resources, mostly being the processor.

The “Transfer_rate” is defined as the portion of available bandwidth used to process an execution unit. This portion includes the input and the output data of the execution unit. The method for quantifying “Transfer_rate” will take into account the characteristics of the interconnecting network. The current transfer rate can be known for each device by monitoring the network. Other methods can be to use historical data in accordance with the operation conditions in each case and situation, or to use averaged data retrieved on regular checks. In many cases, the set of devices P and S are connected to a Wireless Local Area Network (WLAN), and therefore, the latency is reduced and the transfer rate of the network is very often higher than connections through Internet with the cloud servers.

Other components can be defined in order to identify different performance behaviour. For example, ‘Power_consumption’ of the things, ‘Usage_pricing’ of the resources, ‘Security_risk’ of sharing the data, etc.

Every device of the IoT environment, d_i_ ∈ ⅅ, has a performance level, which can be characterized by a function “*Perf*” defined by the following expression:

*Perf*: ⅅ ⟶ 𝕍. (2)

The evaluation of the function *Perf* can be known prior to the execution of the application or can be dynamic over time. For example, a smartphone device will show high values for Response_time if it is busy with user activity. In this case, the performance function must be updated periodically to make the right decisions. Other devices could have stable performance levels due to their dedicated use or their capacity for absorbing new tasks. The static data can be stored in a Look-Up Table (LUT) for fast evaluation of the function.

Once the computational load is characterized with normalized and relative values, each device of the IoT environment shows its ability to run execution units (u) in 𝕌 for the application in a homogeneous and comparable way.

The next section describes how to perform the scheduling of the execution units among the available computing things and remote servers.

### 3.2. IoT Scheduler

The proposed architecture needs an IoT scheduler to distribute the application workload across the available devices of the environment. The aim of this module is to execute the process in a collaborative way taking into account the limitations of the devices and their performance rates.

**Figure 3:**
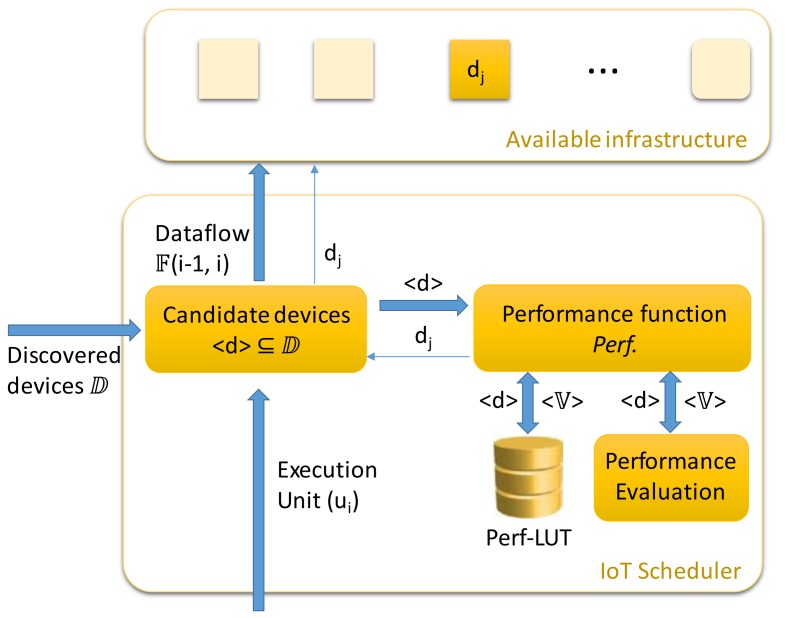
IoT scheduler design.

Usually, the scheduler module of an operating system is a time-consuming process, especially when a complex workload is presented and is needed to make the best choices. In this case, the proposed module is designed to make feasible decisions in the least possible amount of time, even if they are not optimal. The components of this module are shown in Figure 3.

When a new execution unit (u_i_) arrives, it must be evaluated where it should be executed according to the described model. The execution units can be created on the device itself. In any case, the scheduler process is the same. As shown in the previous figure, the IoT scheduler consists of four main components:

(1) The ‘Candidate_devices’ component obtains a list of the available devices that can process the execution unit (u_i_). This list comes from the discovered devices in the system ⅅ. According to the execution unit features, this function selects the feasible devices than can process it.
  Here, a first device filter is introduced, setting up the best chances for processing. The high heterogeneity of IoT resources needs an information model to represent it containing unambiguous and machine-interpretable descriptions of the available resources. In this way, the computing platforms discovered should be described in terms of metadata such as resource type, computing power, memory, location, etc. as well as information to reach its exposed services. If there is a new type of device, then it needs to be registered in the system by cataloguing its features by type of execution unit. Then, when a new execution unit arrives (u_i_), the system knows the set of devices that it is able to compute. Based on this information, this function selects the set of candidate devices.
  When only one device is available for this execution unit, it is selected for processing it. If more than one is listed, it is necessary to decide which to select. The lists of candidate devices are established by default for each type of execution unit according to their capabilities and features.
(2) The ‘Performance_function’ component calculates the *Perf* function for that list of devices. This function determines the best option. For devices with static rates the function can read the data from Perf-LUT. This is a fast operation. Next, if the static data is not available, then it is necessary to estimate or evaluate it for each device by means of an evaluation function. These evaluations can be processed in parallel. Finally, once the performance data is ready, the system chooses the best option. If there are no available devices, the task must to be processed on the device itself or an error flag must be raised.
(3) The ‘Performance_evaluation’ component evaluates or estimates the performance of the selected devices on_the_fly. The delay at this stage depends on the method used. For example, in order to evaluate network performance, a number of multiplatform tools are available. One simple solution is the Iperf (http://iperf.fr) tool. It allows target nodes to be set by running an Iperf process on each platform. As a result, the response time and the transfer rate can be obtained for each available platform. The delay of this calculation can be constrained and then a suboptimal decision is preferable in order to meet the deadline. At this point, other strategies and policies could be addressed, mainly by adapting the successful results from previous and future research. As a result of our previous research on distributed and mobile systems, in [28] a proposal is introduced that combines imprecise computing strategies with cloud computing, which can be used to design a real-time component.
(4) The ‘Perf-LUT’ component stores the static performance data of the devices. It consists of a memory located near the Perf function calculation module with precalculated results for each device and each instance of the <d> vector. The size of this module depends on the knowledge stored about the devices performance.
  When the list of available devices is fixed and the network performance is stable, for example in a controlled environment, the job of the scheduler module is considerably simplified since it can work as a priority list stored in memory. That is, the devices are ordered by features and performance, and then they are selected according to their availability.

## 4. Case Study

In this section, we present a simple case study where the proposed distributed method comes into play to handle an application where sensing and complex computations take place. The aim is to validate our proposal and show the benefits of the architecture in providing flexibility for sharing the processing of the application. The experiments emphasize the advantages of the proposed distributed architecture model, which is more innovative and useful in an IoT environment compared to a traditional centralized model.

The application used as example consists of a method for analyzing attention degree of students in classroom. This information is monitored by the instructor while he/she is giving lessons to the students. In this way, the teacher can know who and when the students are tired or have lost the attention, in a collective and individual way. Please, note that this is only an illustrative example of the advantages of moving the processing load to the edge and promoting the collaborative work among the things around. The added value of this approach mainly falls into emphasize the advantages of the distributed method, being able to test anywhere by professionals, with mobile, tablet or other IoT device.

This problem is largely studied in the field of driving security since the degree of attention of the driver is a key feature to prevent car crashes [65,66,67]. The driver fatigue detectors are including as premium features of high quality cars. These detectors are usually based on analysis of driver facial features such as eyes and yawning. Due to this reason, the typically installed sensor is a digital camera.

**Figure 4:**
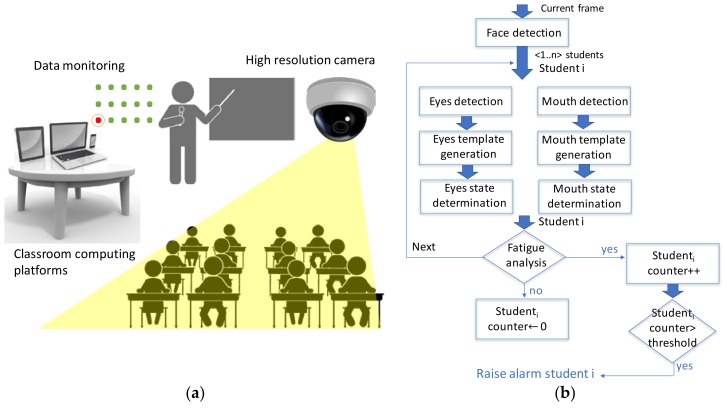
Application scheme: (**a**) Application context inside the classroom; (**b**) Application steps.

Translating this problem to the student attention issue, Figure 4 shows a simple scheme of the application.

Regarding privacy compliance of this application there are two issues that must be met: firstly, consent has to be given since user data (face) is monitored and processed; secondly, the student faces must be processed in an anonymous way. That is, the system can infer when the attention degree of a student is low and raise a flag indicating his/her position in the classroom as shown Figure 4. (red dot), but it cannot identify who is this student, nor relate this with any academic data.

For this application, the list of execution units 𝕌 has two dimensions: It consists of the list of application steps depicted by Figure 4b, and for each student in the classroom. Typically, a school classroom can have around 25 students.

On the other hand, the data flows 𝔽 exchanged between the execution units are frames, frames windows (when the face is located), and data templates. In addition, this application allows a high parallel processing since the eyes and mouth can be processed in parallel, and each student can also be analyzed in parallel. This high parallelism facilitates the collaboration work among different computing platforms.

**Figure 5:**
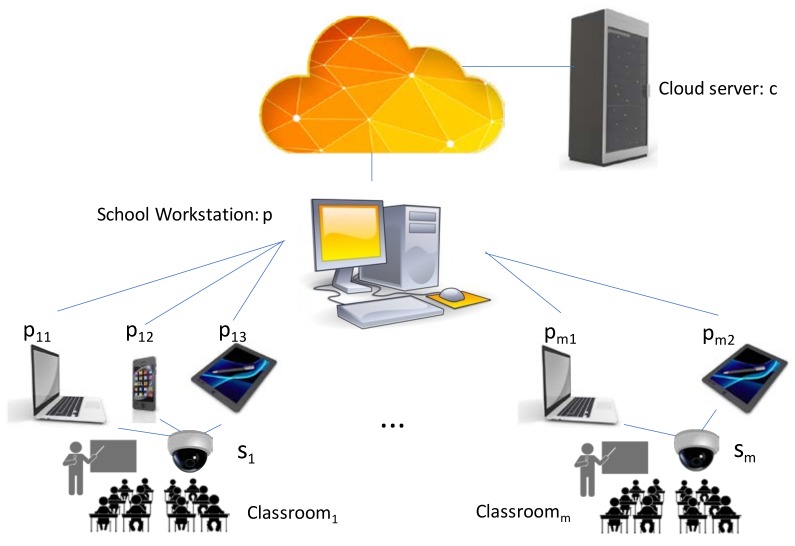
IoT application environment.

The available computing platforms for this application can be deployed at several layers, for example, each classroom is equipped with a mobile PC, and, eventually, the teacher can have a tablet PC and a smartphone that might be involved in the calculations. In addition, the school could have a specific workstation to assist the processing of all classrooms at time, and, in the case of overhead, computing services are hired temporarily from an outside cloud server. A high-resolution camera is employed to provide frames where detecting and searching for several face features of all students at once. The next figure (Figure 5) depicts a scheme of the whole system. This architecture is concretized to the general scheme of the IoT communication network shown in Figure 2a. The value added of the proposed architecture is the collaborative work that takes place under this approach. So that, it does not matter what are the specific distribution of nodes, which can be networked sensors, computing platforms, or cloud servers.

The scheme depicted in Figure 5 shows a possible distribution of the devices where each classroom has a particular set of available devices. For example, Classroom_1_ has three local computing platforms and Classroom_m_ has only two.

Formally, the IoT environment consists of the following devices:

- Sensors set {S} = {s_i_: High resolution camera of classroom i}
- Computing platforms {P} = {p_ij_: computing platform i of classroom j, p: school workstation}
- Cloud server {C} = {c_0_}

The devices of {S} and {P} are in the IoT communications network connected to the school WLAN. The cloud server is accessed through Internet.

Based on the experimentation data of previous works [65], the processing time for the face search in the whole image for a single user is around 0.5 s in a standard workstation. It is supposed the same time for mouth analysis in a frame. Table 2 shows a comparative of the estimated calculation time of a frame for each type of computing platform according to standard hardware configuration of each type of device. It is supposed a classroom with 25 students, and a little school with 12 classrooms. In addition, as shown by Figure 4b, fatigue detection of a student needs a positive search in several consecutive frames. For example, for ‘threshold’ = 5 the costs should be five times longer.

Communication time between platforms within the WLAN are not taken into account in this comparative. For Cloud computing processing an extra delay should be included to transmit the frames (or frame windows). Another drawback arises concerning the security issue when using Cloud resources. In order to meet the new GDPR requirement regarding user data processing, the cloud infrastructure has to be deployed in safe regions that provide an adequate level of data protection [48]. In this way, to keep the processing load of frames inside the WLAN prevents security risks in processing this student behaviour.

The estimative delay times shown by Table 2 give a more precise picture of the situation. The centralized workstation cannot be adopted in many cases due to the long period to detect fatigue in a student. The school could deploy more resource hardware to speed up the calculations. Instead, the proposed IoT architecture can be used to make the most of the existing classroom resources. The combined use of the three computing platforms of the classroom in a collaborative way can drastically reduce the application time. This collaborative work obtains a performance for fatigue analysis application in around 1 min by using only local resources, that is, the classroom mobile PC, Table PC and smartphone by processing all students in parallel. In addition, the whole school can be processed in about the same time by putting all available devices to work together. The school workstation can be used to assist classrooms, centralize all data and infer statistical information about the interests of each lesson.

**Figure 6:**
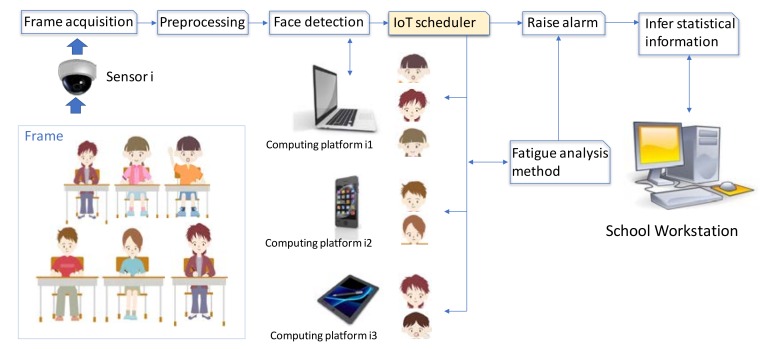
Collaborative work example.

The execution units involved in this application will be responsible for computing a portion of the dataset, for example, the portion of the frame where each student is placed. Figure 6 shows a scheme of this collaborative work.

The scheduler of the IoT architecture can be computed on the classroom mobile PC. Based on the previous features, a possible implementation of the scheduler components for properly handling this IoT application is the following:

- Candidate devices: The instructor can have tablet and/or smartphone able to take part of this application. Thus, this module obtains the list of platforms present on the classroom (classroom mobile PC, table PC, smartphone), the school workstation and the cloud server.
- Performance evaluation module: The performance of the involved devices in this case study is known, and additional devices can not join this application ecosystem in each classroom. Therefore, this function simply obtains the available capacity of every device according to their current workload.
- Perf-LUT: The smartphone and tablet of the instructors are evaluated offline to update this module. At high level, an example of the content of this module is shown in Table 2. More deeply, this LUT stores the computing time of each stage (execution unit) of the method for analyzing the attention degrees of students. The parallelism feature of this application allows the handling of the portion of frame of each student as an execution unit.
- Performance function (Perf): The behaviour of this function can be set to allow the processing as close as possible to where the data are obtained. Therefore, the devices are sorted by proximity and the result retrieved from the performance evaluation module. In general terms, the classroom mobile PC should be selected in the first place, next the tablet PC, and next the smartphone. When these devices are busy, the workload can be outsourced to the centralized workstation and cloud server.

This example shows the possibilities of the proposed architecture to deploy and compute applications in a collaborative way. As shown, the architecture leverages the computing capabilities of available IoT devices to speed up the computations and achieve better performance than in a centralized way. In addition, this alternative could not be available due to network congestions, or privacy issues.

For an accurate deployment, a calibration step is needed of the available objects that could take part in the processing, as well as a further application analysis for building the graph of executions units and data flows. This information helps to configure the Performance Evaluation Module of the scheduler. In addition, the application requirements and the working environment constraints play an important role in designing the collaborative network and proper profiling the scheduler.

The implementation of the execution units would also have to take into account limited-resource target devices such as wearables, sensing devices and other mobile computers. In these cases, some key features would be considered such as the enhanced communication capabilities and battery consumption (if applicable).

## 5. Conclusions

In this paper, a wide review of the state of the art of distributed computing for IoT systems is described. It is a common issue that computing requirements for monitoring and advanced analysing of the data acquired by IoT environments usually exceed the capabilities of the sensors and even the mobile computers. In this work, a distributed architecture that combines sensing and processing at different levels of the network to share the computing load among the available devices has been proposed to address this challenge. The IoT environments composed of wearables and other biosensors may benefit from this architecture by allowing the processing of advanced applications with real-time constraints in a collaborative way.

Besides summarizing the relevant and representative contributions, as well as the background of IoT distributed computing, the research presented in this paper represents a further step for designing collaborative schemes of IoT applications. The main advantage of the proposed system is that it enables real-time monitoring and analysis of all the acquired data by networked devices. In addition, it provides flexibility in executing the application by using resources from cloud computing services, but also from other available computers that are recommendable for their cost reduction or better availability. The key novelties are the application formalization and its decomposition in execution units, and the new scheduler module. This is the core component of the architecture to distribute the execution units and dispatch them according to the computing capabilities of each device.

A simplified case study is included to demonstrate that our approach is possible to implement in any area. In this case, we have used an educational environment as IoT is drastically changing the education around the world. We would like to emphasize that the aim of this case study is just to present our general model and particularized to the IoT.

Further work must be invested in building a proper predictive model of the available resources. System resilience is another interesting issue that should be further developed. The proposed framework could support a fallback policy, to be activated when central control is not available or accessible. This point could be expanded by adding a service discovery mechanism, allowing computers to autonomously take suboptimal decisions based on local information coming from neighbouring devices. Currently, a larger amount of data is being gathered, including open data, in order to construct a more challenging experimental scenario.

## Figures and Tables

**Table 1:** Recent advances on IoT distributed computing.

Research Line	Main Contribution Area
(i) *Framework design for distributed computing*	
Managing the quality of experience in the multimedia IoT [16]	Quality of Experience
QoS-Aware scheduling of services-oriented IoT [17]	Scheduling method
Distributed computational model for shared processing [18]	Distributed computing model
IoT-Based Computational Framework [19]	Distributed computing model
A scalable IoT framework using virtual sensor [20]	Virtual sensor framework
Middleware for Internet of Things [22]	Middleware
MinT: Middleware for Cooperative Interaction of Things [23]	Middleware
Integration of Edge, IoT and the Cloud [24]	Edge of Things
Scheduling internet of things Apps in cloud computing [25]	Scheduling method
Payload-size and deadline-aware scheduling [26]	Scheduling method
Task Requirement Aware Pre-processing and Scheduling [27]	Scheduling method
Flexible framework for real-time embedded systems [28]	Scheduling method
(ii) *Integration with Cloud Computing resources*	
IoT and Cloud Computing [29]	General analysis
Machine learning for IoT [5]	Cloud-based Intelligence
Model of Internet of Things and Cloud (IoT-Cloud) [30]	Mobile cloud computing
A study on cloud-based Internet of Things: CloudIoT [31]	General analysis
Integration of Cloud computing and IoT [32]	Survey
Cloud Computing and Internet of Things Integration [33]	General analysis
Framework for computation offloading [34]	Mobile Cloud Computing
MCC for computation offloading [35]	Mobile Cloud Computing
Multi-Criteria Decision Analysis Methods [36]	Offloading process analysis
Stochastic Analysis of Delayed Mobile Offloading [37]	Offloading process analysis
Application-oriented offloading [38]	Offloading process analysis
Mobile Cloud Services [39]	Mobile Cloud Services
(iii) *Security*	
Trust computation models for service management in IoT [40]	Survey
Secure integration of IoT and Cloud Computing [41]	IoT-Cloud security
Security and privacy challenges in MCC [42]	MCC security
Security, privacy and trust in IoT [43]	Survey
Cyber security framework for IoT-based Energy Internet [44]	Intelligent Security System
Fog computing security [49]	Fog computing security
Distributed intrusion detection system [50]	Distributed system security
GDPR and the Internet of Things [46]	GDPR
Normative challenges of identification [47]	GDPR
(iv) *Distributed applications design*	
Design flow for web service applications [51]	Model-based design
The web of things [52]	Web service -based design
Cloud-based platform for distributed IoT applications [53]	Deployment platform
Commercial frameworks for the IoT [54]	Survey of design platforms
A Self-Managing Containerized IoT Platform [55]	Design platform
IoT Design Patterns [56]	Design patterns
Data Mining proposal of distributed applications events [57]	Data Mining
Open IoT Ecosystem [58]	Deployment platform
Future Internet of Things Controller [59]	Decentralized Intelligence
IoT and Multiagent Systems [60]	Decentralized Intelligence

**Table 2:** Time estimation of fatigue analysis application.

Computing Platform	Frame Computing Cost	Threshold = 5
Classroom Mobile PC ^1^	25 s	~2 min
Classroom Tablet PC ^1^	50 s	~4 min
Classroom Smartphone ^1^	50 s	~4 min
School Workstation ^2^	5 min	25 min
Classroom resources ^1^	13 s	~1 min
Cloud Server ^3^	25 s + 5 s	2.5 min

^1^ Total time for 25 students. ^2^ Total time for 12 classrooms of 25 students. ^3^ Total time for 12 classrooms of 25 students plus communications delay.
